# Prevalence of post-traumatic stress disorder symptoms among patients with mental disorder during the COVID-19 pandemic

**DOI:** 10.1186/s12888-022-03790-w

**Published:** 2022-03-01

**Authors:** Lirong Tang, Yue Gao, Shuangyi Qi, Jie Cui, Li Zhou, Yi Feng

**Affiliations:** 1 Department of Clinical Psychology Center, Beijing Anding Hospital, Capital Medical University, Beijing, China; 2 The National Clinical Research Center for Mental Disorders & Beijing Key Laboratory of Mental Disorders, Beijing Anding Hospital, Capital Medical University, Beijing, China; 3 Advanced Innovation Center for Human Brain Protection, Capital Medical University, Beijing, China; 4 Mental Health Center, Central University of Finance and Economics, 39 South College Road, Haidian District, 100081 Beijing, China; 5 Faculty of Psychology, Beijing Normal University, Beijing, China

**Keywords:** Post-traumatic stress disorder, Patients with mental disorder, Mental disorder, Associated factors, COVID-19

## Abstract

**Background:**

The outbreak of the COVID-19 pandemic has caused extensive public health concerns, posing significant challenges to healthcare services. One particular area of concern is the mental health of patients with mental disorder, who are often a neglected group. The aim of this study was to investigate the prevalence of, and associated factors for symptoms of post-traumatic stress disorder (PTSD) among patients with mental disorder in China during the COVID-19 pandemic.

**Methods:**

Self-reported questionnaires were distributed to patients in four psychiatric hospitals in Beijing, China, between April 28th and May 30th, 2020. Information regarding sociodemographic characteristics, COVID-19 related factors, support, psychosomatic factors, and PTSD symptoms were collected using a series of scales, such as the Impact of Event Scale-Revised, the 7-item Generalized Anxiety Disorder Scale, the 9-item Patient Health Questionnaire depression scale, and so on. Multivariate regression was used to identify factors related to PTSD symptoms.

**Results:**

A total of 1,055 patients with mental disorder were included in the final sample. The prevalence of PTSD symptoms was 41.3%. Hierarchical linear regression demonstrated that fear of the pandemic and anxiety were shared associated factors for both symptoms of PTSD and their subscales. Additionally, age was an associated factor for the total PTSD (*β* = 0.12, *p* < 0.01), intrusion (*β* = 0.18, *p* < 0.001), and avoidance (*β* = 0.1, *p* < 0.05) symptoms; depression was an associated factor for the total PTSD (*β* = 0.13, *p* < 0.001), intrusion (*β* = 0.11, *p* < 0.01), and hyperarousal (*β* = 0.19, *p* < 0.001) symptoms.

**Conclusions:**

The prevalence of PTSD symptoms was high among patients with mental disorder during the COVID-19 pandemic in China. This study found that age, fear of the pandemic, anxiety and depression are significant associated factors of PTSD symptoms in patients with mental disorder during the pandemic. We call for higher awareness and introduction of PTSD interventions to relieve the psychological stress in these patients.

## Background

The COVID-19 pandemic has had a substantial impact on many aspects of the physical and mental health of the population worldwide [1]. Psychiatric symptoms have been increasing in both the general population and in patients with the infection during the pandemic [2–4]. Patients with mental disorder, who are often a neglected group, have also encountered mental health problems during the pandemic, even if not infected with COVID-19 [5]. Patients with mental disorder, including affective and schizophrenia spectrum disorders, are at a higher risk of negative mental health outcomes related to the pandemic [6]. There are indications of worsening psychiatric symptoms among patients with pre-existing mental disorders [7, 8]. Some experts have speculated that the COVID-19 pandemic might be negatively affecting individuals with pre-existing mental disorders [9–11]. With a 16.6% lifetime prevalence of mental disorders among adults in China [12], millions of patients with mental disorder need to be concerned, as they may face barriers when seeking help and timely management of their mental health condition during the pandemic [13]. However, little appears to be known about the pandemic’s impact on patients with pre-existing mental disorders [14].

Post-traumatic stress disorder (PTSD) is caused by exposure to actual or threatened death, serious injury or sexual violence [15]. There are three main types of symptoms: intrusion symptoms associated with the traumatic events (such as intrusive memories, recurrent distressing dreams, intense or prolonged psychological distress, dissociative reactions, and marked physiological reactions), persistent avoidance symptoms (including avoidance of distressing memories, thoughts or feelings, and numbing of responsiveness), and hyperarousal symptoms (including irritable behavior, anger outbursts, problems with concentration, hypervigilance, and exaggerated startle response) [16]. Individuals with PTSD are generally at higher risk of suicide [17]. Long-term exposure to stress may worsen pre-existing chronic health conditions, accelerate the progression of the disease, or increase the financial burden on patients [18]. Some experts consider PTSD as a secondary effect of the pandemic [17], during which many people are reporting numbness, stiffness, high vigilance, and other psychiatric symptoms [18]. Studies on COVID-19 revealed that PTSD could occur during and after infectious diseases [19]. The prevalence of PTSD symptoms ranged from 7 to 53.8% in the general population during the COVID-19 pandemic in China, Spain, Italy, Iran, the US, Turkey, Nepal, and Denmark [20]. A meta-analysis including 68 independent samples and sub-samples indicated that the PTSD prevalence was 21.94% during the COVID-19 pandemic, and pandemic-affected groups have significantly higher PTSD prevalence compared to the general population under normal circumstances [21]. A systematic review of the relationship between the COVID-19 pandemic and mental health consequences found that mental health issues in COVID-19 infected patients presented a high level of post-traumatic stress symptoms (96.2%) [14]. Previous psychiatric disorders displayed suggestive evidence of increasing the risk of PTSD [22]. The onset of PTSD symptoms can make the psychiatric disorder itself more complex and difficult to treat, leading to a greater disease burden [23]. Therefore, clinical doctors need to increase the awareness on the importance of PTSD symptoms in patients with mental disorder. However, until now there has been no research on the prevalence of PTSD symptoms among patients with mental disorder during the COVID-19 pandemic.

The causes of PTSD are not fully understood, and whether people who have experienced the same traumatic event develop PTSD is related to sociodemographic characteristics and pre -, peri-, and post-traumatic factors, which interact in complex ways [22]. A systematic review of 54 studies on PTSD found that six pre-traumatic predictors of PTSD included: cognitive level, coping styles; personality characteristics, psychopathology, psychophysiological factors, and socio-ecological factors [24]. Variables related to coping strategies and social/family support showed evidence as PTSD associated factors [22, 25]. All potential consequences of trauma (i.e., symptoms of anxiety, avoidance, or depression) had evidence as post-trauma risk factors [22]. In previous literature, a number of risk and protective factors for PTSD have been identified, however, these findings have not always been consistent [26], inconsistency may reflect unrecognized or unaccounted sources of genuine heterogeneity or biases.

The aim of this study was to examine the prevalence of PTSD symptoms among patients with mental disorder during the COVID-19 pandemic, and to identify associated factors for PTSD symptoms and their subscales. We assumed that due to their susceptibility and vulnerability to crises, the prevalence of PTSD symptoms among patients with mental disorder might be higher than that of the general population during the pandemic [10, 11]. Based on previous study, in addition to sociodemographic characteristics and COVID-19-related factors, we used psychosomatic factors from the perspective of psychological factors (i.e., loneliness, anxiety, and depression), somatic factors (i.e., quality of life, sleep quality), and social ecological factors (i.e., social support) as possible associated factors for PTSD symptoms [24, 27]. Above all, we hypothesized as follows: (1) The prevalence of PTSD symptoms among patients with mental disorder will be higher than that among general population during the pandemic. (2) Demographic characteristics of the patients with mental disorder, such as age and gender, will be significantly associated with the PTSD symptoms. (3) COVID-19-related factors, such as fear of the pandemic and the increased pressure by pandemic, will be associated factors with PTSD symptoms. (4) Psychosomatic factors (i.e., loneliness, quality of life, sleep quality, anxiety, and depression) will significantly associate with PTSD symptoms among patients with mental disorder.

## Methods

### Participants and Procedures

This cross-sectional survey was conducted from April 28 to May 30, 2020. Cluster sampling was used to construct the sample. A questionnaire was distributed by three psychiatrists to all patients from four psychiatric hospitals located in different districts of Beijing, China. The three psychiatrists came from four different hospitals, one of whom worked in two hospitals. The inclusion criteria for participants were as follows: able to write; aged 18–60 years; diagnosed with anxiety disorder, major depressive disorder, bipolar disorder, or schizophrenia on his/her medical record before this survey. All participants were informed about the purpose of the study and its procedures before taking part in it. They were also informed that they could refuse to answer any item and withdraw at any time during the study. Each participant signed an informed consent form before completing the questionnaire. 1,104 patients participated in this study, 49 of whom did not complete the questionnaire and were excluded, leaving the data of 1,055 participants available for analysis, with a completion rate of 95.6%. The study protocol was performed in accordance with the Declaration of Helsinki [28]. The Ethics Committee of Beijing Anding Hospital affiliated to Capital Medical University has approved this investigation.

### Measures

#### Sociodemographic Characteristics

Sociodemographic data was collected, including sex, age, education level, marital status, employment status, annual family income, place of residence, medication status during the pandemic, substance use, and living circumstance during the pandemic. Information was also collected on psychiatric diagnoses, including anxiety disorder, major depressive disorder, bipolar disorder, and schizophrenia.

Medication status during the pandemic was recorded as one of the following three types: on psychotropic medications, on non-psychotropic medications, and not on any medications. Substance use was assessed through two questions: “During last month, have you ever experienced symptoms of intoxication such as dizziness, headache and drowsiness due to drinking too much?” on a 5-point scale (1 = *never*; 2 = *rarely*; 3 = *occasionally*; 4 = *ofte*n; 5 = *almost every day*); and “How many cigarettes did you smoke per day on average?” on a 5-point scale (1 = *No smoking history*; 2 = *Smoking 1-5 cigarettes per day*; 3 = *5-10 cigarettes*; 4 =*10-20 cigarettes*; 5 = *more than 20 cigarettes per day*), with higher scores indicating more severe substance use. The final score was obtained by summing the items.

#### PTSD Symptoms

PTSD symptoms were measured using the 22-item self-reported Impact of Event Scale-Revised (IES-R) [16], which is used to assess the severity of subjective distress caused by traumatic events. Its items are categorised into three symptom subdomains, namely intrusion, avoidance, and hyperarousal, each of which is rated on a 5-point scale (0 = *not at all*; 4 = *extremely*). The IES-R has been validated, including in China [29], for research regarding health-related trauma and associated with severe acute respiratory syndromes [30, 31]. We considered a cut-off score of 24 for the present study based on previous research [16]. We calculated a composite score for PTSD (Cronbach’s α = 0.93) and its symptom subscales (Cronbach’s α = 0.84–0.84).

#### COVID-19-related Factors

COVID-19-related factors included fear of the pandemic, increased mental pressure by pandemic, seeking clinical treatment during pandemic, seeking mental health guidance during pandemic, and medication barriers caused by the pandemic. All the five factors were measured through self-designed questions, due to the limited questionnaire related to COVID-19 pandemic in previous studies.

Fear of the pandemic was assessed by two questions: “In the last week, were you worried about getting infected with COVID-19?” and “Do you wash your hands excessively for fear of getting infected with the virus?” both on a 5-point scale (1 = *never*; 2 = *rarely*; 3 = *occasionally*; 4 = *often*; 5 = *almost every day*), with higher scores indicating stronger concern. The final values were obtained by the average score. Increased mental pressure by pandemic was assessed by two questions: “Is your medication status greatly affected by the outbreak? “, and “Did your mental state fluctuate during the outbreak?” both on a 5-point scale with higher scores indicating much less affection and fluctuation. Seeking clinical treatment during the pandemic was assessed by one question: “Did you see a psychiatrist during the outbreak?” requiring a “*yes*” or “*no*” answer. Seeking mental health guidance during pandemic was assessed by one question: “Did you receive mental health services during the outbreak?”, again with a *yes* or *no* answer possibility. Medication barriers due to pandemic were also assessed by one question: “Has your access to medicines been affected during the outbreak?” on a 6-point scale, with answers ranging from 1 = *never* to 6 = *very often*, with higher scores representing more severe medication barriers.

#### Psychosomatic Factors

Psychosomatic factors include loneliness, quality of life, sleep quality, anxiety, and depression in the present study.

Anxiety was evaluated using the 7-item Generalized Anxiety Disorder Scale (GAD-7) [32], which assesses the frequency of anxiety symptoms in the past two weeks on a 4-point scale (0 = *not at all*; 3 = *nearly every day*). The Chinese version of the GAD-7 has been validated (α = 0.90), and we considered 5 as the clinical cut-off score based on previous research. Cronbach’s α was 0.95 in the present study.

Depressive symptoms were measured by the 9-item Patient Health Questionnaire (PHQ-9) [33], which assesses the frequency of depressive symptoms in the past two weeks on a 4-point scale (0 = *none*; 1 = *on a few days*; 2 = *on more than half of the days*; 3 = *almost every day*). The Chinese version of the PHQ-9 has been validated (Cronbach’s α = 0.94) [34], and we considered 5 as the clinical cut-off score based on previous research [35].

Loneliness was assessed by the question “Do you feel lonely?” on a 5-point scale (1 = *never*; 2 = *rarely*; 3 = *occasionally*; 4 = *often*; 5 = *almost every day*), with higher scores representing more severe loneliness. Quality of life was assessed by two questions: “How do you feel about your quality of life?” and “Are you satisfied with your present state of health?” on a 5-point scale (1 = *extremely unsatisfactory*; 2 = *rarely not satisfied*; 3 = *not satisfied or dissatisfied*; 4 = *very satisfied*; 5 = *extremely satisfactory*), with higher scores indicating a higher quality of life and a greater satisfaction. We obtained the final score by summing the items, with Cronbach’s α of 0.76. Sleep quality was assessed by the following three items according to the Pittsburgh Sleep Questionnaire (PSQI-PT) [36]: “How many hours of sleep have you usually been getting per night during the pandemic?”, “Do you take medication to help you sleep during the pandemic?”, and “In general, what do you think of your ability to sleep during the pandemic?” The responses were evaluated using a 5-point scale, with higher scores indicating a worse quality of sleep. We obtained a composite score by adding the items, with Cronbach’s α of 0.56.

#### Support

Support was assessed by six items adapted from the Multi-Dimensional Scale of Perceived Social Support (MSPSS) [37], which has been validated in China [38]. Participants were asked to rate the agreement of six items during the pandemic on a 5-point scale (1 = *strongly disagree*; 2 = *disagree*; 3 = *not sure*; 4 = *agree*; 5 = *strongly agree*). For example, “There is a special person who is around when I am in need during the COVID-19 pandemic”, “I get the emotional help and support I need from my family during the COVID-19 pandemic”, and “During the COVID-19 pandemic, I have friends with whom I can share my joys and sorrows”. Each two questions represent three subscales (i.e., social support from significant others, family support, and friends support) respectively. A composite score of support was calculated by summing up six items (Cronbach’s α = 0.79). Three composite scores for subscales were also calculated by summing up corresponding two items respectively (Cronbach’s α = 0.75–0.87).

### Data Analysis

The characteristics of the sample are presented as mean ± standard deviation for continuous variables, and as the percentage for categorical variables. Hierarchical linear regression models were used to identify factors related to the PTSD symptom subscales. In Step 1, we entered the sociodemographic variables into the model, before adding COVID-19 related factors in Step 2. In Step 3, we added support and in Step 4, we included psychosomatic factors. All categorical variables (e.g., sex, employment, and diagnosis, etc.) were coded as dummy variables in the regression models. An increasing *R*^2^ value further confirmed the importance of the independent variables regarding the dependent ones. One-way variance analysis and post-hoc tests were used to evaluate PTSD symptoms according to the underlying diagnosis. The statistical analyses were performed using the IBM SPSS Statistics software version 20.0 and R software version 3.6.1. All tests were two-tailed. A *p*-value less than 0.05 was considered statistically significant.

## Results

### Sociodemographic and Clinical Characteristics

A total of 1,055 patients with mental disorder entered into the formal analysis. Of these, 506 (48.0%) participants were from a specialized tertiary hospital, 276 (26.2%) were from a second-level psychiatric hospital, and 273 (25.9%) were from two community psychiatric hospitals. As shown in Table 1, the average age of the participants was 37.15 (*SD* = 13.21) years. The characteristics that represented a majority of the participants were as follows: female (65.5%), a lower education level (62.2%), unmarried/others (52.6%), living in an urban area (88.4%), annual family income lower than 150,000 CNY (71.9%), and not infected with COVID-19 (93.9%). All participants had a pre-existing diagnosis (35.4% with anxiety disorder, 26.7% with major depressive disorder, 17.6% with bipolar disorder, and 20.3% with schizophrenia). During the COVID-19 pandemic, almost half of these patients reported not seeking mental health services (47.7%). More than half of the participants were taking psychiatric medicine (57.3%) but reported significant COVID-19-related barriers to continuing treatment.

**Table 1:** Demographic and clinical characteristics of the study sample (*N* = 1,055)

Variables	Number	Percent (*%*)
*Mean* Age *(SD)*	37.15	(13.21)
Sex		
Male	364	34.5
Female	691	65.5
Education level		
Junior school or lower	183	17.3
High school	474	44.9
College or above	398	37.7
Employment status		
Full-time	419	39.7
Part-time	61	5.8
Unemployed	283	26.8
Retired	142	13.5
Student	150	14.2
Marital status		
Married	500	47.4
Unmarried	455	43.1
Others/Not clear	100	9.5
Family annual income		
< 30,000	253	24.0
30,000 ~ 60,000	210	19.9
60,000 ~150,000	295	28.0
150,000 ~250,000	152	14.4
> 250,000	145	13.7
Residence place		
Urban	933	88.4
Rural	122	11.6
Residence status during the pandemic		
Live alone	110	10.4
Live together with others	878	83.2
Live in hospital	62	5.9
Others	5	0.5
Infection with COVID-19		
No	991	93.9
Yes	64	6.1
*Mean* Substance use *(SD)*	1.36	(0.66)
Medication status during pandemic		
On psychotropic medications (yes)	604	57.3
Not on any medications (no)	214	20.3
On non-psychotropic medications (no)	237	22.5
Mental disorder diagnosis		
Anxiety disorder	373	35.4
Major depressive disorder	282	26.7
Bipolar disorder	186	17.6
Schizophrenia	214	20.3
*Mean* Fear of pandemic *(SD)*	2.46	(0.67)
*Mean* Increased pressure by pandemic *(SD)*	2.23	(0.93)
Psychiatric treatment during pandemic		
Yes	652	61.8
No	403	38.2
Mental health guidance during pandemic		
Yes	552	52.3
No	503	47.7
*Mean* Medication barrier during pandemic *(SD)*	2.15	(1.02)
Supports		
*Mean* Social support *(SD)*	4.43	(1.34)
*Mean* Family support *(SD)*	4.98	(1.48)
*Mean* Friends support *(SD)*	4.71	(1.41)
Psychosomatic factors		
*Mean* Loneliness *(SD)*	2.39	(1.31)
*Mean* Quality of life *(SD)*	3.04	(0.89)
*Mean* Sleep quality *(SD)*	2.43	(0.88)
Generalized anxiety symptoms (≥5)	540	51.0
Depressive symptoms (≥5)	604	57.3
PTSD symptoms (≥24)	436	41.3

*Note*. The unit of annual income is CNY yuan. Categorical variables were presented in the form of mean and standardized deviation, and continuous variables were presented in the form of number and proportion

### Prevalence of PTSD Symptoms

**Fig. 1:**
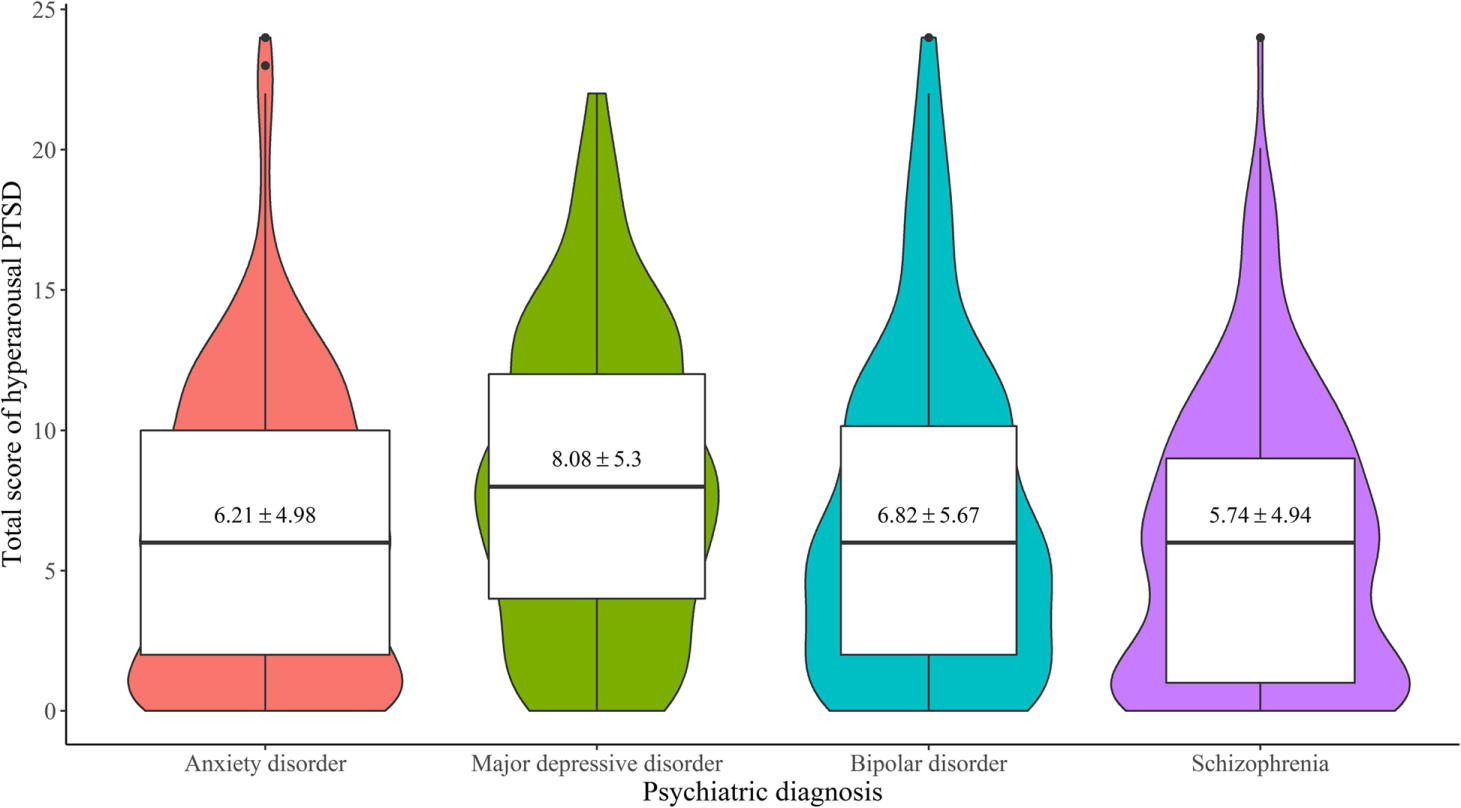
Total scores of hyperarousal symptom of PTSD among patients with different mental disorder diagnosis. *Note.* The width of the figures indicates the sample size of each group

436 patients (41.3%) had PTSD symptoms, which were then investigated according to underlying psychiatric diagnosis (anxiety disorder, major depressive disorder, bipolar disorder, or schizophrenia). There was no significant between-group difference in the total score of PTSD (*F* (3, 1049) = 2.22, *p* = 0.084), the intrusion symptom (*F* (3,1051) = 1.16, *p* = 0.324), or the avoidance symptom (*F* (3,1051) = 0.394, *p* = 0.758). Patients with major depressive disorder had the highest mean score (14.08 ± 5.30) for the hyperarousal symptom, significantly higher than the anxiety group (*p* < 0.05) and the schizophrenia group (*p* < 0.05) (see Fig. 1).

### Factors Associated with PTSD Symptoms

As shown in Table 2, the hierarchical linear regression demonstrated that fear of the pandemic and anxiety were shared associated factors for both PTSD symptoms and their subscales. Additionally, age was an associated factor for total PTSD score (*β* = 0.12, *p* < 0.01), intrusion (*β* = 0.18, *p* < 0.001), and avoidance (*β* = 0.1, *p* < 0.05); depressive symptoms were associated factors for total PTSD score (*β* = 0.13, *p* < 0.001), intrusion (*β* = 0.11, *p* < 0.01), and hyperarousal (*β* = 0.19, *p* < 0.001); retirement was a shared associated factor for both the total PTSD score (*β* =-0.07, *p* < 0.05) and intrusion (*β* = - 0.07, *p* < 0.01); mental health guidance during the pandemic was a unique associated factor for PTSD symptoms (*β* = - 0.05, *p* < 0.05); medication status during the pandemic (*β*= - 0.08*p* < 0.05) and psychiatric treatment (*β* = 0.06, *p* < 0.05) were unique associated factors for intrusion; quality of life was a unique associated factor for avoidance (*β* = 0.09, *p* < 0.05); urban residence (*β* = 0.04, *p* < 0.05), increased pressure (*β* = 0.09, *p* < 0.05), family support (*β* = 0.05, *p* < 0.05), friends support (*β*= - 0.05, *p* < 0.05), loneliness (*β* = 0.06, *p* < 0.05), and sleep quality (*β* = 0.06, *p* < 0.01) were all unique associated factors for hyperarousal.

**Table 2:** Hierarchical linear regression coefficients for different PTSD symptoms (*N* = 1,055)

Variables	PTSD				Intrusion subscale				Avoidance subscale				Hyperarousal subscale			
	Model 1	Model 2	Model 3	Model 4	Model 1	Model 2	Model 3	Model 4	Model 1	Model 2	Model 3	Model 4	Model 1	Model 2	Model 3	Model 4
***Socio-demographics characteristics***																
Sex (male)	-0.05	-0.00	-0.01	0.01	-0.03	0.02	0.02	0.03	-0.04	-0.01	-0.01	0.00	-0.07*	-0.02	-0.02	0.00
Age	-0.00	0.03	0.05	0.12**	0.08	0.10*	0.16*	0.18***	0.02	0.04	0.05	0.10*	-0.11*	-0.06	-0.04	0.03
Education	-0.04	-0.02	-0.00	0.01	-0.02	0.00	0.01	0.03	-0.04	-0.02	-0.01	0.00	-0.04	-0.03	-0.01	0.00
Employment (part-time)	-0.08*	-0.06*	-0.06*	-0.03	-0.06	-0.05	-0.05	-0.03	-0.06	-0.05	-0.06	-0.03	-0.08*	-0.06*	-0.06*	-0.02
Employment (unemployed)	-0.04	-0.01	-0.03	-0.00	-0.06	-0.03	-0.04	-0.03	-0.02	0.01	-0.00	0.02	-0.04	-0.01	-0.03	-0.00
Employment (retired)	-0.06	-0.07	-0.08	-0.07*	-0.06	-0.07	-0.07	-0.07*	-0.06	-0.07	-0.07	-0.07	-0.03	-0.05	-0.05	-0.04
Employment (student)	-0.08	-0.05	-0.05	-0.04	-0.07	-0.04	-0.04	-0.03	-0.06	-0.04	-0.04	-0.03	-0.09*	-0.06	-0.06	-0.05
Marital status (unmarried)	-0.03	-0.00	-0.01	0.01	-0.04	-0.02	-0.03	-0.00	-0.00	0.02	0.01	0.03	-0.03	-0.01	-0.02	-0.00
Marital status (others)	0.01	0.03	0.03	0.00	-0.04	-0.01	-0.01	-0.03	0.05	0.07*	0.07*	0.05	-0.00	0.01	0.01	-0.02
Income	-0.02	0.01	0.01	-0.04	-0.01	0.01	0.02	-0.02	-0.04	-0.03	-0.03	-0.05	0.01	0.04	0.04	-0.01
Residence (urban)	0.03	0.03	0.03	0.03	-0.01	-0.01	-0.01	-0.01	0.05	0.05	0.05	0.05	0.04	0.04	0.04	0.04*
Live (alone)	0.00	0.01	0.00	0.02	0.05	0.02	0.01	0.02	0.01	0.02	0.01	0.02	-0.02	-0.01	-0.02	0.00
Live (hospital)	0.01	-0.00	-0.01	-0.00	0.04	0.02	0.02	0.02	-0.00	-0.02	-0.02	-0.02	-0.01	-0.01	-0.02	-0.01
Live (others)	-0.06*	-0.06	-0.05	-0.03	-0.05	-0.04	-0.03	-0.02	-0.07*	-0.06	-0.05	-0.05	-0.05	-0.04	-0.03	-0.02
Infection (yes)	0.02	-0.01	-0.01	0.01	0.02	0.01	0.00	0.01	0.01	0.00	0.00	0.01	0.00	-0.02	-0.02	0.01
Substance use	0.14***	0.06*	0.05	-0.01	0.11**	0.05	0.04	-0.01	0.12***	0.06	0.05	0.01	0.15***	0.06	0.05	-0.02
Medication (yes)	-0.00	-0.05	-0.03	-0.05	-0.03	-0.08*	-0.06	-0.08*	0.01	-0.03	-0.02	-0.03	0.02	-0.03	-0.01	-0.04
Diagnosis (anxiety)	0.04	0.06	0.05	0.05	0.02	0.03	0.03	0.02	0.06	0.07	0.06	0.07	0.04	0.04	0.03	0.02
Diagnosis (depression)	0.11**	0.07	0.04	-0.04	0.10	0.06	0.04	-0.03	0.04	0.01	-0.01	-0.05	0.18***	0.12**	0.09*	-0.02
Diagnosis (bipolar)	0.07	0.01	-0.00	0.01	0.06*	0.01	0.00	0.01	0.05	0.01	0.00	0.01	0.07	-0.00	-0.01	-0.01
***COVID-19-related factors***																
Fear of pandemic		0.27***	0.27***	0.20***		0.31***	0.31***	0.25***		0.22***	0.22***	0.17***		0.19***	0.19***	0.11***
Increased pressure		0.37***	0.32***	0.04		0.27***	0.24***	0.01		0.23***	0.20***	0.02		0.50***	0.44***	0.09*
Psychiatric treatment		0.06	0.06	0.05		0.07*	0.07*	0.06*		0.05	0.05	0.05		0.03	0.04	0.03
Mental health guidance		-0.01	-0.03	-0.05*		0.01	-0.00	-0.03		-0.06	-0.07*	-0.08**		0.03	0.01	-0.02
Medication barrier		-0.14**	-0.12**	0.03		-0.10*	-0.09	0.03		-0.06	-0.05	0.04		-0.22***	-0.20***	-0.01
***Support***																
Social support			-0.04	-0.04			-0.02	-0.02			-0.05	-0.05			-0.04	-0.03
Family support			-0.10**	0.01			-0.11**	-0.02			-0.07	-0.00			-0.09**	0.05*
Friends support			-0.09*	-0.03			-0.05	-0.00			-0.05	-0.02			-0.16***	-0.05*
***Psychosomatic factors***																
Loneliness				0.05				0.01				0.05				0.06*
Quality of life				0.05				0.06				0.09*				-0.03
Sleep quality				0.04				0.05				0.00				0.06**
Anxiety				0.47***				0.41***				0.35***				0.51***
Depression				0.13**				0.11*				0.06				0.19***
***Adjusted R***^***2***^	0.02	0.18	0.21	0.45	0.02	0.17	0.19	0.36	0.01	0.10	0.12	0.23	0.05	0.21	0.26	0.62
***△R***^***2***^	0.02**	0.16***	0.04***	0.24***	0.02**	0.15***	0.02***	0.17***	0.01*	0.09***	0.02***	0.11***	0.05***	0.16***	0.05***	0.35***

*Note*. All the regression coefficients in the above table were standardized regression coefficients. All categorical variables were coded as dummy variables in the regression models. Sociodemographic variables (e.g., sex, age, etc.) were entered in Model 1; COVID-19 related factors (e.g., fear of pandemic, increased pressure, etc.) were entered in Model 2; Support (i.e., social, family and friends support) were entered in Model 3; Psychosomatic factors (e.g., Loneliness, sleep quality, etc.) were entered in Model 4. *p < 0.05; **p < 0.01; ***p < 0.001

## Discussion

The prevalence of PTSD symptoms (41.3%) in this study is much higher than the previously reported lifetime prevalence level in general population (2–9%) [39]. A systematic review showed that the pooled prevalence of PTSD among participants during the COVID-19 pandemic was 21.94% [21]. A study conducted between April 4 and 6, 2020, among the medics working in Wuhan upon their return after work indicated an overall prevalence of clinically concerned PTSD symptoms of 31.6% [40], by the same scale (IES-R). A survey-based cross-sectional study performed from January 29 to February 7, 2020 in China showed an estimated PTSD prevalence of 9.8% among healthcare workers who worked in hospitals with fever clinics or wards for COVID-19 infected patients [41]. In our study, this prevalence of PTSD was even higher. Exact comparison is difficult to make because some of the other research have used different measures. However, it is suggested that although Beijing is not an area with the highest risk of COVID-19, the prevalence of PTSD symptoms in patients with mental disorder in Beijing is high, indicating the susceptibility of this group. Another possible explanation for the increased prevalence of PTSD may be due to the decreased treatment adherence of patients with mental disorders during the COVID-19 pandemic [42–44]. Clinicians must be aware that these patients may experience higher rates and severity of post-traumatic stress disorder than general population [20].

In this study, there was no significant difference between the severity of the total PTSD score among patients with different mental disorder diagnosis. This may indicate that patients with different diseases share similar psychological characteristics, including vulnerability and susceptibility, causing similar effects during the COVID-19 pandemic. However, the score of the PTSD hyperarousal symptoms was higher in patients with major depressive disorder than in those with anxiety disorder or schizophrenia. Evidence suggests that the associations between PTSD and depression are complex, involving bidirectional causality, common risk factors, and common vulnerabilities [23, 45, 46]. Hyperarousal includes irritability, anger, difficulty in concentrating, hypervigilance, and a heightened startle response [16]. The results of this study suggest that more attention should be paid to the characteristics of high arousal in patients with major depressive disorder.

The study found evidence for the second hypothesis that demographic characteristics were associated with PTSD symptoms. This study showed that age was an associated factor for the total PTSD score, intrusion, and avoidance. Since the COVID-19 virus is more serious and has a higher mortality rate in older people [47], they may have more severe PTSD. Retirement was a shared associated factor for both the total PTSD score and intrusion in the study, indicating that retirement may be a protective factor for PTSD. A possible explanation is that retirees may need to travel less during the epidemic and have a higher financial security, therefore being less stressed by the epidemic [1].

The third hypothesis, COVID-19 related factors are associated with PTSD symptoms, was well supported by the data. Fear of the pandemic was a shared associated factor for both PTSD symptoms and their subscales. There have been reports that anxiety and fear often co-exist and comorbid with PTSD [48, 49]. Mental health guidance during the pandemic was a unique associated factor, while clinical treatment during the pandemic, or medication barriers due to the pandemic was not significantly associated factor for PTSD symptoms, which might indicate that mental health interventions and resources could help patients with mental disorder reduce the stress caused by the epidemic and the incidence of PTSD.

The data supported the fourth hypothesis, that is, psychosomatic factors are significant associated factors with PTSD symptoms. Anxiety symptoms were shared as associated factors for both PTSD symptoms and their subscales. Recent neuroscience research suggested that higher sensitivity to anxiety tended to increase the severity of PTSD [50]. Individuals with higher stress/fear levels might become impatient, feel upset or agitated, and experience difficulty relaxing, all of which would have a negative impact on PTSD symptoms [51]. Depression symptoms were associated factors for the total PTSD score, intrusion and hyperarousal. As depression is the disorder most commonly associated with PTSD [23, 46], people with depressive symptoms may be more likely to develop PTSD, which should be particularly noticed. Quality of life was a unique associated factor for avoidance, implying that patients were more concerned about it. During the epidemic, people’s quality of life deteriorated [52]. According to a study in China, self-rated poor health during an outbreak was significantly associated with a greater psychological impact and higher levels of stress [31].

Another prominent finding was that several unique factors were associated with sub-dimensions of PTSD. Most obviously, urban residence, increased pressure, loneliness, support from friends and sleep quality were all unique associated factor for hyperarousal but not associated with intrusion or avoidance. These results might indicate that there were differences among the related factors of the three dimensions of PTSD, and hyperarousal required unique attention [16]. During an epidemic, isolation policies and inadequate social support can lead to feelings of loneliness [1]. Previous studies showed that isolation could negatively affect mental health [7, 8]. Our findings correlate to those of other studies on general population. Social support plays a key role in mitigating the risk of mental health problems [53]. The results also demonstrated that support from friends was associated with a lower incidence of hyperarousal symptoms, while support from family might increase patients’ hyperarousal symptoms. This finding is a reminder that too much unnecessary care from family could increase patients’ hyperarousal symptoms. Thus, “moderate” care from friends is necessary for patients with mental disorder. These results have great implications for clinicians in predicting and treating patients with high hyperarousal symptoms.

### Implications

To the best of our knowledge, this is the first study to screen for PTSD symptoms in patients with a pre-existing mental disorder diagnosis during the COVID-19 pandemic in Beijing, China. Primarily, the prevalence of PTSD symptoms among patients with mental disorder was not encouraging, arousing attention from medical staff, related psychologists and mental health centers. Next, this study explored some risk factors (e.g., old age, depressive disorder, fear) and protective factors (e.g., retirement, mental health guidance) for PTSD, providing a specific reference and guidance for the psychological prevention and intervention among patients with mental disorder in the face of the COVID-19 pandemic. Furthermore, this study examined PTSD as well as the three subscales, discriminating the difference in the relationship between PTSD subscales and related psychosomatic factors. The uniqueness of the hyperarousal factor provided a theoretical reference for better understanding the structure of PTSD symptoms.

### Limitations

This study has several limitations that should be considered when interpreting its findings. First, it adopted a cross-sectional design, so it is unclear how PTSD symptoms in patients with mental disorder might change over time. A longitudinal study is required to identify protective factors and the long-term impact of PTSD in patients with mental disorder during the pandemic. Second, the sample was limited to patients from just four psychiatric hospitals in Beijing, China. Therefore, a nationwide or worldwide multicentre study is needed to provide broader data about PTSD symptoms among patients with mental disorder during the COVID-19 pandemic. Finally, no objective biological indicators were included as psychosomatic factors. In further research, other indicators such as peripheral blood, heredity, inflammation, immune and metabolic function markers, or brain imaging are necessary.

## Conclusions

This study found that the prevalence of PTSD symptoms was high among patients with mental disorder during the COVID-19 pandemic in China, as well as the associated factors for PTSD symptoms, including socio-demographic and psychosomatic factors, shedding practical implication on the PTSD status among patients with mental disorder. We recommended that clinical psychiatrists increase the awareness of PTSD symptoms among patients with mental disorder and provide effective mental health interventions for them to manage those symptoms.

## Data Availability

The datasets used during the current study are available from the corresponding author on reasonable request.
